# Ferroptosis and Nrf2 Signaling in Head and Neck Cancer: Resistance Mechanisms and Therapeutic Prospects

**DOI:** 10.3390/antiox14080993

**Published:** 2025-08-13

**Authors:** Jaewang Lee, Youngin Seo, Jong-Lyel Roh

**Affiliations:** 1 Department of Otorhinolaryngology-Head and Neck Surgery, CHA Bundang Medical Center, CHA University, Seongnam 13488, Republic of Korea; 2 Logsynk, Seoul 06153, Republic of Korea; 3 College of Medicine, Gyeongsang National University, Jinju 52828, Republic of Korea; yseo7777@naver.com; 4 Department of Biomedical Science, General Graduate School, CHA University, Pocheon 11160, Republic of Korea

**Keywords:** ferroptosis, nuclear factor erythroid 2-related factor 2, head and neck cancer, therapy, resistance

## Abstract

Ferroptosis is an iron-dependent form of regulated cell death marked by lipid peroxidation in polyunsaturated phospholipids. In head and neck cancer (HNC), where resistance to chemotherapy and immunotherapy is common, ferroptosis offers a mechanistically distinct strategy to overcome therapeutic failure. However, cancer cells often evade ferroptosis via activation of nuclear factor erythroid 2-related factor 2 (Nrf2), a key regulator of antioxidant and iron-regulatory genes. HNC remains therapeutically challenging due to therapy resistance driven by redox adaptation. This review highlights the ferroptosis pathway—a form of regulated necrosis driven by iron and lipid peroxidation—and its regulation by Nrf2, a master antioxidant transcription factor. We detail how Nrf2 contributes to ferroptosis evasion in HNC and summarize emerging preclinical studies targeting this axis. The review aims to synthesize molecular insights and propose therapeutic perspectives for overcoming resistance in HNC by modulating Nrf2–ferroptosis signaling. We conducted a structured narrative review of the literature using PubMed databases. Relevant studies from 2015 to 2025 focusing on ferroptosis, Nrf2 signaling, and head and neck cancer were selected based on their experimental design, novelty, and relevance to clinical resistance mechanisms. In HNC, Nrf2 mediates resistance through transcriptional upregulation of GPX4 and SLC7A11, epigenetic stabilization by PRMT4 and ALKBH5, and activation by FGF5 and platelet-derived extracellular vesicles. Epstein–Barr virus (EBV) infection also enhances Nrf2 signaling in nasopharyngeal carcinoma. More recently, loss-of-function KEAP1 mutations have been linked to persistent Nrf2 activation and upregulation of NQO1, which confer resistance to both ferroptosis and immune checkpoint therapy. Targeting NQO1 in KEAP1-deficient models restores ferroptosis and reactivates antitumor immunity. Additionally, the natural alkaloid trigonelline has shown promise in reversing Nrf2-mediated ferroptosis resistance in cisplatin-refractory tumors. Pharmacologic agents such as auranofin, fucoxanthin, carnosic acid, and disulfiram/copper complexes have demonstrated efficacy in sensitizing HNC to ferroptosis by disrupting the Nrf2 axis. This review summarizes emerging mechanisms of ferroptosis evasion and highlights therapeutic strategies targeting the Nrf2–ferroptosis network. Integrating ferroptosis inducers with immune and chemotherapeutic approaches may provide new opportunities for overcoming resistance in head and neck malignancies.

## 1. Introduction

Ferroptosis has emerged as a distinct form of regulated cell death defined by iron-catalyzed lipid peroxidation and the collapse of antioxidant defense systems, particularly those involving glutathione (GSH) and glutathione peroxidase 4 (GPX4) activity [1,2,3]. Unlike apoptosis or necroptosis, ferroptosis is driven by the accumulation of lethal lipid peroxides, especially within polyunsaturated fatty acid-containing phospholipids, and it is strongly influenced by iron availability and redox homeostasis [3,4]. Recent studies have established the therapeutic relevance of ferroptosis in targeting malignant tumors that exhibit resistance to conventional treatment modalities [5,6,7]. However, cancer cells have developed adaptive mechanisms to counteract oxidative stress, often mediated through metabolic rewiring and transcriptional activation of protective genes [8,9,10].

At the core of this adaptive resistance lies nuclear factor erythroid 2-related factor 2 (Nrf2, encoded by the *NFE2L2*), a redox-sensitive transcription factor that orchestrates the expression of cytoprotective genes via antioxidant response elements (AREs) [11]. Under non-stressed conditions, Nrf2 is tightly regulated by its inhibitor Kelch-like ECH-associated protein 1 (Keap1), which targets it for proteasomal degradation [12]. However, upon oxidative stress or electrophilic insults, the Keap1-Nrf2 complex is disrupted, leading to Nrf2 stabilization, nuclear translocation, and transcriptional activation of numerous genes that combat oxidative damage, regulate iron metabolism, and suppress lipid peroxidation [13,14]. While this response preserves cellular integrity in normal tissues, persistent Nrf2 activation contributes to treatment resistance in cancer cells, including protection from ferroptosis-induced cell death [15,16]. The Nrf2–Keap1 pathway regulates the cellular antioxidant response and is constitutively activated in many cancer types, including HNC. This constitutive activation enhances antioxidant capacity, enabling cancer cells to evade ROS-mediated cell death and promoting chemo- and radioresistance [17,18,19]. This connection between oxidative stress and cell death is further supported by studies demonstrating that Nrf2 not only modulates antioxidant capacity but also directly limits ferroptosis by mitigating lipid peroxidation through its downstream targets, including GPX4, solute carrier family 7 member 11 (SLC7A11), and heme oxygenase-1 (HO-1) [20]. These findings highlight the Nrf2–lipid peroxidation–ferroptosis axis as a pivotal regulatory node in redox-sensitive pathologies, including cancer. Beyond cancer, NRF2/KEAP1 signaling is critically involved in the pathogenesis of several chronic diseases, including neurodegeneration, metabolic syndrome, and cardiovascular disease, highlighting its dual role in cytoprotection and disease progression [21,22,23].

In the context of head and neck cancers (HNCs), including head and neck squamous cell carcinoma (HNSCC), which arises from the mucosal linings of the oral cavity, nasal cavity, paranasal sinus, pharynx, and larynx, head and neck cancers (HNCs) account for 1,464,550 new cases and 487,993 deaths according to the Global Cancer Statistics of 2020, posing a profound impact on health worldwide [24]. The majority are squamous cell carcinomas (HNSCCs), which are commonly linked to tobacco, alcohol, and human papillomavirus. Despite advances in surgery, radiation, and immunotherapy, treatment failures due to radio- and chemoresistance remain common and are associated with oxidative stress adaptation mechanisms [25]. Accumulating evidence supports a critical role for ferroptosis and its modulation by Nrf2 in determining therapeutic outcomes [26]. These malignancies are frequently characterized by elevated oxidative stress and metabolic plasticity, yet they paradoxically evade ferroptosis due to the upregulation of antioxidant and iron-buffering systems under the control of Nrf2 [27,28]. For example, cisplatin-resistant HNC cells exhibit heightened expression of SLC7A11 and GPX4, both of which are transcriptional targets of Nrf2, and contribute to the evasion of lipid peroxidation-induced death [27]. Functional studies have shown that suppression of Nrf2 using siRNA or small-molecule inhibitors re-sensitizes these resistant cells to ferroptotic agents such as RSL3 and artesunate, highlighting the centrality of the Nrf2–GPX4 axis in modulating drug resistance [27,28].

Moreover, recent reports have identified several upstream and downstream regulators of Nrf2 that further enhance ferroptosis resistance in HNC. For instance, overexpression of AlkB homolog 5, RNA demethylase (ALKBH5) stabilizes *NFE2L2* mRNA via *N*^6^-methyladenosine (m^6^A) demethylation and insulin-like growth factor 2 mRNA-binding protein 2 (IGF2BP2) binding, leading to increased resistance to ferroptosis in hypopharyngeal HNSCC [29]. In nasopharyngeal carcinoma (NPC), cancer-associated fibroblast-derived *fibroblast growth factor 5* (FGF5) activates *fibroblast growth factor receptor 2* (FGFR2)–Nrf2 signaling, thereby dampening cisplatin-induced ferroptosis and promoting chemoresistance [30]. Similarly, platelet-derived extracellular vesicles have been shown to transfer integrin β3, activating Nrf2 signaling through mitogen-activated protein kinase (MAPK)/*activating transcription factor 4* (ATF4) pathways and supporting ferroptosis evasion in NPC cells [31]. These findings underscore the diverse mechanisms through which Nrf2 integrates extrinsic and intrinsic signals to regulate redox homeostasis and ferroptotic sensitivity in head and neck tumors.

Given these insights, the therapeutic manipulation of ferroptosis through inhibition of Nrf2 and its network has gained attention as a strategy to overcome drug resistance and augment immunotherapy in HNC. In particular, targeting upstream activators such as *protein arginine N-methyltransferase-4* (PRMT4), *thioredoxin reductase 1* (TXNRD1), and Keap1 mutations, as well as exploring combination strategies with ferroptosis inducers and immune checkpoint inhibitors, may unlock new avenues in cancer treatment [32,33,34]. In this review, we aim to provide an updated and comprehensive analysis of the ferroptosis–Nrf2 axis in HNC. By integrating recent findings from molecular, preclinical, and translational studies, we examine how Nrf2 modulates ferroptosis sensitivity, contributes to treatment resistance, and represents a viable target for novel therapeutic interventions in HNC. Compared to existing reviews, this article uniquely focuses on the interface between Nrf2-driven antioxidant signaling and ferroptosis regulation, specifically in HNC. It highlights recent studies addressing ferroptosis sensitivity in the context of Nrf2 activation and therapy resistance, providing a focused narrative for translational applications.

## 2. Molecular Interplay Between Ferroptosis and Nrf2 Signaling

Ferroptosis is a form of regulated cell death that is mechanistically distinct from apoptosis and necroptosis, characterized by iron-dependent accumulation of lipid peroxides within polyunsaturated phospholipids [35,36]. The core biochemical events driving ferroptosis include the elevation of intracellular ferrous iron (Fe^2+^), which catalyzes reactive oxygen species (ROS) generation via Fenton reactions, and the depletion or dysfunction of antioxidant systems such as the GSH–GPX4 axis [37]. Once cellular lipid peroxidation exceeds the detoxification capacity of GPX4, membrane integrity is compromised, resulting in cell death [38]. This mechanism is particularly relevant in malignant cells with altered metabolic and redox states [7].

**Figure 1:**
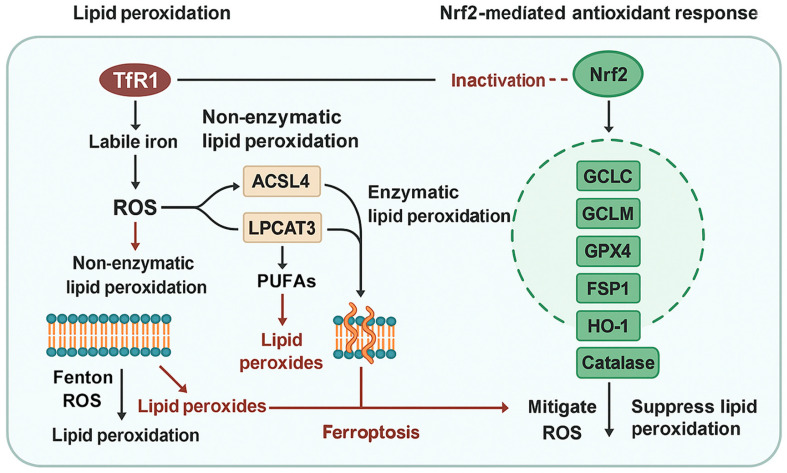
Molecular crosstalk between lipid peroxidation and Nrf2-mediated antioxidant defense in ferroptosis regulation. Transferrin receptor 1 (TfR1)-mediated iron uptake increases labile iron, which catalyzes reactive oxygen species (ROS) production and drives both non-enzymatic and enzymatic lipid peroxidation. Polyunsaturated fatty acids (PUFAs) are activated by ACSL4 and incorporated into membrane phospholipids via LPCAT3, making them susceptible to lipid peroxidation and ferroptosis. The accumulation of lipid peroxides compromises membrane integrity, culminating in ferroptotic cell death. In contrast, Nrf2 activation induces a transcriptional program that enhances cellular antioxidant capacity through upregulation of genes such as GCLC, GCLM, GPX4, FSP1, HO-1, and catalase. These genes mitigate ROS accumulation, suppress lipid peroxidation, and buffer labile iron, collectively counteracting ferroptosis. In head and neck cancer, sustained Nrf2 activation promotes ferroptosis resistance and supports tumor survival under oxidative and metabolic stress.

Nrf2 plays a critical role in defending cells from oxidative and electrophilic stress by regulating the expression of a wide spectrum of antioxidant genes. Upon activation, Nrf2 translocates into the nucleus and binds to AREs in the promoters of its target genes, many of which directly influence ferroptosis susceptibility [39]. Among these are SLC7A11, encoding the cystine/glutamate antiporter system Xc^−^, which imports cystine for GSH synthesis, and GPX4, which catalyzes the reduction of lipid hydroperoxides [40]. The upregulation of these genes collectively enhances the capacity of cancer cells to neutralize ROS and lipid peroxides, thereby conferring resistance to ferroptosis (Figure 1) [20,41].

Beyond these canonical targets, Nrf2 also regulates genes involved in iron storage and trafficking, including ferritin heavy chain 1 (FTH1) and ferroportin (FPN1) [42]. These factors help sequester or export redox-active iron, limiting the iron-catalyzed formation of hydroxyl radicals that initiate lipid peroxidation [43]. Additionally, HO-1, another Nrf2 target, plays a dual role; while it degrades pro-oxidant heme into biliverdin, iron, and carbon monoxide, the liberated iron can paradoxically contribute to ferroptosis under certain conditions [44]. However, in most cancer settings, the net effect of HO-1 overexpression is cytoprotective, particularly when accompanied by ferritin-mediated iron sequestration [45].

Beyond its role in GSH metabolism, Nrf2 also tightly regulates intracellular iron dynamics through transcriptional control of ferritin, FPN1, and HO-1 [42]. These factors coordinate iron sequestration, storage, and release, thereby modulating the labile iron pool (LIP). Interestingly, while increased HO-1 activity may enhance free iron availability, which can promote ferroptosis, the concomitant induction of ferritin by Nrf2 buffers this effect, limiting ROS propagation [46,47]. In HNC models, this iron-buffering function appears to outweigh pro-ferroptotic signals, as sustained Nrf2 activation is associated with ferroptosis suppression despite elevated HO-1 expression [30,48]. These findings highlight a tightly regulated balance, wherein Nrf2 adjusts both oxidative stress and iron overload, favoring cell survival in malignancy [49,50].

Importantly, in head and neck cancer models, activation of the Nrf2 signaling pathway has been repeatedly associated with decreased ferroptosis sensitivity. For example, in cisplatin-resistant HNC cells, increased expression of p62 (SQSTM1, sequestosome-1) leads to Keap1 inactivation, resulting in sustained Nrf2 activation and transcription of SLC7A11 and GPX4 [27]. These alterations enhance GSH biosynthesis and lipid peroxide detoxification, thereby preventing ferroptotic cell death induced by erastin or RSL3 [27,28]. Genetic or pharmacologic inhibition of Nrf2 restores ferroptotic sensitivity in these models, underscoring its central regulatory function.

Furthermore, recent studies in NPC have demonstrated that exogenous factors from the tumor microenvironment also influence the Nrf2–ferroptosis axis. For instance, FGF5 derived from cancer-associated fibroblasts can activate FGFR2 on tumor cells, initiating downstream Keap1/Nrf2/HO-1 signaling and enhancing resistance to ferroptosis [30]. Similarly, extracellular vesicles originating from platelets can transfer *integrin subunit beta 3* (ITGB3) to NPC cells, where it activates the MAPK–ATF4–Nrf2 cascade and suppresses ferroptosis through stabilization of SLC7A11 [31].

These findings highlight the intricate regulation of ferroptosis by Nrf2, which not only governs cellular redox capacity but also integrates environmental cues and oncogenic signals to enhance tumor cell survival. In HNC, where oxidative stress is inherently elevated due to metabolic reprogramming and therapy-induced ROS, Nrf2-mediated reinforcement of ferroptosis resistance provides a critical advantage for tumor progression and therapy evasion [41,51]. Targeting this regulatory axis—either by direct inhibition of Nrf2 or through blockade of its upstream activators and downstream effectors—offers a compelling opportunity to disrupt redox homeostasis and re-sensitize cancer cells to ferroptosis-inducing agents.

## 3. The Role of Nrf2 in Ferroptosis Evasion in Head and Neck Cancer

Head and neck cancers (HNCs), encompassing oral, nasopharyngeal, hypopharyngeal, and laryngeal malignancies, are characterized by high oxidative stress and frequent treatment resistance [52,53]. Among the emerging mechanisms that contribute to this resistance is the ability of tumor cells to suppress ferroptosis—a regulated, iron-dependent form of non-apoptotic cell death [7]. Central to this suppression is the activation of the Nrf2 pathway, which modulates multiple antioxidant, metabolic, and iron-regulating processes that collectively protect against ferroptotic triggers [51,54].

**Figure 2:**
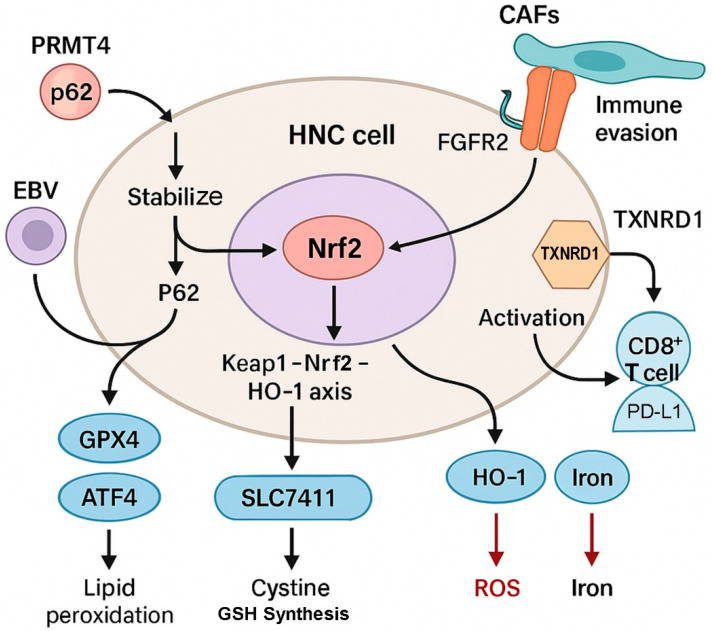
The role of Nrf2 in ferroptosis evasion in head and neck cancer. This diagram illustrates how multiple inputs—including EBV, PRMT4, p62 accumulation, and fibroblast-derived FGF5—activate Nrf2 signaling in HNC cells. Nrf2 promotes the expression of downstream targets such as SLC7A11, GPX4, and HO-1, enabling resistance to lipid peroxidation and iron-dependent ferroptotic cell death. TXNRD1 further supports this axis by stabilizing redox balance and promoting immune evasion via PD-L1 expression, ultimately impairing CD8^+^ T-cell-mediated ferroptosis. These cooperative mechanisms highlight Nrf2’s central role in redox resilience and therapeutic resistance.

Recent experimental evidence in HNC models has demonstrated that aberrant Nrf2 signaling is a major determinant of resistance to ferroptosis inducers such as GPX4 inhibitors and ROS-amplifying agents (Figure 2). In one study, Shin et al. revealed that cisplatin-resistant and RSL3-resistant HNC cells exhibited upregulation of Nrf2 and downstream antioxidant defenses [28]. These cells also showed increased expression of p62 and activation of the protein kinase R-like ER kinase (PERK)–ATF4–sestrin 2 (SESN2) pathway, which collectively contributed to ferroptosis resistance. Genetic or pharmacologic suppression of Nrf2 restored ferroptosis sensitivity, highlighting the critical role of this pathway in regulating redox resilience in HNC cells.

Compared to lung cancer and glioblastoma (GBM), which often rely on SLC7A11 and GPX4 overexpression for ferroptosis evasion, HNC appears to exhibit distinct ferroptosis resistance patterns through Nrf2-mediated transcriptional upregulation of FSP1, HO-1, and lipid metabolic enzymes [15]. Furthermore, HNC shows differential iron metabolism and antioxidant pathway reliance, suggesting tissue-specific ferroptosis vulnerabilities.

Similarly, Roh et al. demonstrated that artesunate-induced ferroptosis was markedly attenuated in cisplatin-resistant HNC cells due to activation of the Nrf2–ARE transcriptional axis [27]. The upregulation of SLC7A11, GPX4, and HO-1 played a protective role in limiting lipid peroxidation. However, co-treatment with Nrf2 inhibitors or siRNA targeting p62 reversed this resistance and re-sensitized resistant cells to artesunate in vitro and in vivo, validating the mechanistic importance of Nrf2 stabilization in evading ferroptosis-mediated death [27].

The tumor microenvironment in HNC also influences ferroptosis susceptibility through Nrf2-mediated pathways. Cancer-associated fibroblasts (CAFs), for instance, secrete FGF5, which binds to FGFR2 on NPC cells and activates the Keap1–Nrf2–HO-1 axis. This signaling cascade protects tumor cells from cisplatin-induced ferroptosis, as shown by Liu et al. in an NPC xenograft model [30]. The presence of CAF-derived FGF5 not only inhibited lipid peroxidation but also diminished therapeutic efficacy, underscoring the role of stromal signals in ferroptosis evasion via Nrf2 activation.

Another mechanism of Nrf2-driven ferroptosis resistance involves the upregulation of TXNRD1, as recently reported by Hsieh et al. TXNRD1 overexpression maintained cellular redox balance and suppressed ferroptosis while simultaneously promoting programmed death-ligand 1 (PD-L1) expression and immune evasion in HNSCC [33]. Inhibition of TXNRD1 using auranofin not only disrupted Nrf2 signaling but also sensitized tumors to immune checkpoint inhibitors (anti-PD-1 therapy) by enhancing CD8^+^ T-cell-mediated ferroptosis. These findings identify a critical immunometabolic axis—TXNRD1–Nrf2–PD-L1—that mediates both ferroptosis resistance and immune checkpoint blockade failure in HNSCC.

PRMT4 has emerged as another upstream regulator of Nrf2 that supports ferroptosis evasion in NPC. Pu et al. showed that PRMT4 upregulation in cisplatin-resistant NPC cells enhanced Nrf2 and GPX4 expression, thereby attenuating erastin-induced ferroptosis [32]. Silencing of PRMT4 or pharmacologic inhibition of Nrf2 restored mitochondrial damage and lipid peroxidation, leading to significant tumor suppression in mouse models. These data suggest that the PRMT4–Nrf2–GPX4 axis may be a viable therapeutic target to overcome ferroptosis resistance in NPC.

Further complicating the ferroptosis landscape in HNC is the involvement of virus-mediated modulation. In Epstein–Barr virus (EBV)-positive NPC cells, EBV infection was found to stabilize Nrf2 via the p62–Keap1–Nrf2 pathway, leading to elevated SLC7A11 and GPX4 expression [55]. These changes suppressed ferroptosis and promoted resistance to chemotherapeutic agents. Knockdown of GPX4 or pharmacologic inhibition of this pathway re-sensitized EBV-infected NPC cells to ferroptosis, suggesting viral involvement in reinforcing Nrf2-mediated antioxidant defenses.

Antioxidant enzymes such as GPX4, SOD, catalase, and paraoxonase-2 (PON2) play pivotal roles in maintaining redox homeostasis and preventing ferroptosis. PON2 has been found to be upregulated in HNC and oral squamous cell carcinoma (OSCC), contributing to resistance against cisplatin and enhancing tumor survival under oxidative stress [56,57,58]. Silencing of PON2 sensitizes cancer cells to chemotherapy by increasing lipid peroxidation, suggesting its potential as a ferroptosis-sensitizing target.

The cumulative effect of these findings indicates that Nrf2 operates at the intersection of metabolic plasticity, redox control, and therapy resistance in head and neck malignancies. By orchestrating the expression of detoxifying enzymes (e.g., GPX4 and HO-1), cystine transporters (e.g., SLC7A11), iron regulators, and immune modulators (e.g., PD-L1), Nrf2 enables tumor cells to adapt to ferroptotic stress. This makes the Nrf2 signaling axis an attractive but complex target for therapeutic intervention. Targeting Nrf2 directly remains challenging due to its role in maintaining normal cell function under physiological stress [59]. However, selective disruption of its downstream effectors, such as TXNRD1, PRMT4, or specific transport systems, may provide opportunities to sensitize tumors to ferroptosis without systemic toxicity [60,61]. Furthermore, combinatorial strategies using ferroptosis inducers alongside Nrf2 antagonists or immunotherapies offer promising avenues to overcome resistance and improve clinical outcomes in HNC patients [16,51].

## 4. Mechanistic Determinants of Ferroptosis Resistance in Head and Neck Cancer

**Figure 3:**
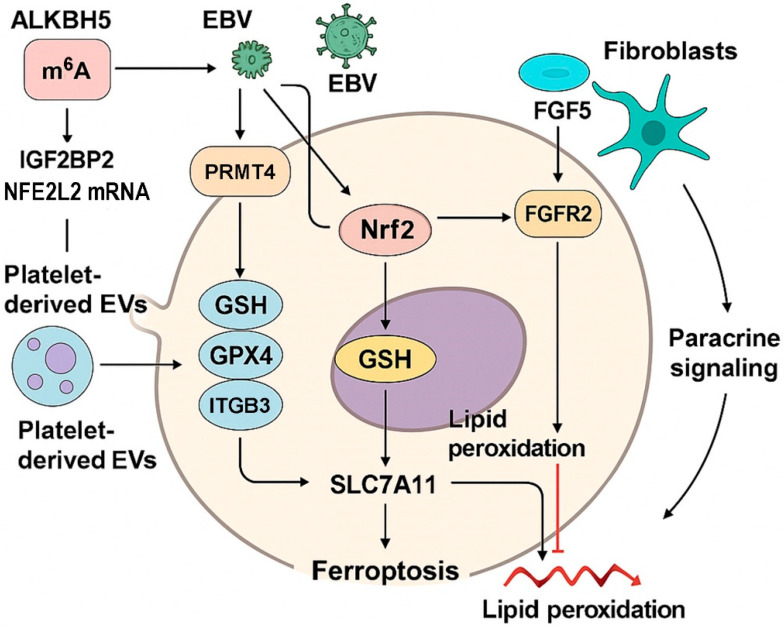
Mechanistic determinants of ferroptosis resistance in head and neck cancer. This figure illustrates converging molecular pathways that suppress ferroptosis in HNC. ALKBH5 reduces m^6^A methylation on NFE2L2 mRNA, promoting stabilization via IGF2BP2. EBV infection activates the p62–Keap1–Nrf2 axis and promotes PRMT4-mediated stabilization of Nrf2. Platelet-derived EVs transfer ITGB3, activating MAPK–ATF4 signaling and increasing SLC7A11 expression. Fibroblast-secreted FGF5 stimulates FGFR2, leading to HO-1 and Nrf2 activation. These pathways converge on the Nrf2–SLC7A11–GSH–GPX4 axis, reducing lipid peroxidation and enabling tumor cells to evade ferroptosis.

Before exploring therapeutic approaches to induce ferroptosis, it is critical to understand the molecular underpinnings that enable HNC to evade this regulated form of cell death. Multiple resistance mechanisms have been identified across several HNC subtypes. These mechanisms span post-transcriptional RNA modifications, epigenetic stabilization of redox regulators, viral reprogramming, and paracrine signaling within the tumor microenvironment (Figure 3). At the center of this resistance network lies the Nrf2 pathway, which orchestrates antioxidant defense, lipid peroxide detoxification, and iron metabolism. This section systematically examines the endogenous and exogenous molecular circuits that converge on the Nrf2–SLC7A11–GPX4 axis, ultimately suppressing ferroptosis and contributing to treatment resistance in HNC.

### 4.1. RNA Epigenetic Regulation and the m^6^A Machinery

RNA methylation has emerged as a key determinant of transcript stability, particularly under oxidative stress. In hypopharyngeal squamous cell carcinoma (SCC), ALKBH5-mediated demethylation of NFE2L2 mRNA decreases m^6^A modification at its 3’-UTR, enabling the m^6^A reader IGF2BP2 to bind and stabilize the transcript [29]. This post-transcriptional regulation enhances Nrf2 expression and promotes resistance to ferroptosis. Ye et al. showed that knocking down ALKBH5 disrupted this axis, reduced Nrf2 protein levels, and sensitized hypopharyngeal SCC cells to ferroptotic death.

### 4.2. Oncoviral Reprogramming via the Nrf2 Pathway

Virus-associated HNCs, especially EBV-positive NPC, exhibit heightened ferroptosis resistance due to viral modulation of host redox systems. Yuan et al. demonstrated that EBV upregulated the p62–Keap1–Nrf2 axis, leading to increased expression of SLC7A11 and GPX4, thereby suppressing lipid peroxidation [55]. Disruption of this pathway restored ferroptosis sensitivity, implicating the EBV–Nrf2 interaction as a critical therapeutic target in virally driven tumors.

### 4.3. Platelet-Derived Extracellular Vesicles and Intercellular Crosstalk

Tumor–platelet interactions have been shown to promote metastasis and drug resistance. In NPC, platelet-derived extracellular vesicles (EVs) enriched in integrin β3 (ITGB3) transferred this integrin into tumor cells, activating the MAPK/ERK/ATF4/Nrf2 axis [31]. This cascade led to increased SLC7A11 expression and decreased lipid peroxidation, thereby suppressing ferroptosis. Li et al. showed that interfering with EV uptake or blocking downstream signaling partially restored ferroptosis sensitivity and reduced metastatic potential.

### 4.4. Epigenetic Stabilization of Nrf2 by PRMT4

Post-translational modifications, particularly protein methylation, also regulate ferroptosis resistance. In cisplatin-resistant NPC cells, PRMT4 was found to interact with and methylate Nrf2, preventing its ubiquitin-mediated degradation [32]. This stabilization upregulated GPX4 and SLC7A11, contributing to resistance. Pu et al. showed that inhibiting PRMT4 sensitized cells to ferroptosis-inducing agents like erastin and reduced tumor growth in vivo.

### 4.5. Paracrine Activation by Cancer-Associated Fibroblasts

The tumor microenvironment exerts a profound influence on ferroptosis sensitivity. Liu et al. reported that fibroblast-derived FGF5 in the NPC microenvironment activated FGFR2 on tumor cells, stimulating the Keap1–Nrf2–HO-1 axis [30]. This paracrine signaling protected cells from cisplatin-induced ferroptosis. Blocking FGF5 or FGFR2 sensitized tumors to oxidative stress, suggesting that microenvironmental cues are integral to ferroptosis resistance.

### 4.6. The SLC7A11–GSH–GPX4 Axis in Redox Compensation

SLC7A11, a direct transcriptional target of Nrf2, imports cystine to support GSH synthesis and lipid peroxide detoxification [41,62]. Its overexpression is a hallmark of multiple resistant HNC subtypes. In EBV-infected and PRMT4-overexpressing models, SLC7A11 knockdown restored ferroptosis, validating its role as a downstream effector in the ferroptosis-resistant phenotype [32,55].

### 4.7. Thioredoxin Reductase 1 (TXNRD1) and Immune Evasion

In HNSCC, TXNRD1 maintains redox balance and supports immune evasion through Nrf2 stabilization [33]. Auranofin-mediated inhibition of TXNRD1 triggers ferroptosis, downregulates PD-L1, and enhances CD8+ T-cell infiltration. These dual effects suggest that TXNRD1 is both a redox regulator and an immune checkpoint modulator. Furthermore, ATF4, which is activated downstream of MAPK, cooperates with Nrf2 to drive SLC7A11 expression under nutrient stress, reinforcing ferroptosis resistance and immune escape [28,31].

### 4.8. KEAP1 Mutation, NQO1 Upregulation, and Ferroptosis–Immune Resistance Axis

In addition to upstream Nrf2 activators and post-transcriptional regulators, recent evidence highlights the significance of KEAP1 mutations in conferring resistance to ferroptosis and immunotherapy in HNC [63,64]. KEAP1 loss-of-function mutations lead to constitutive activation of Nrf2 and upregulation of its downstream targets, including NAD(P)H quinone dehydrogenase 1 (NQO1) [65]. Yuan et al. demonstrated that KEAP1-deficient HNSCC models exhibit ferroptosis resistance and impaired antitumor immune responses [34]. However, pharmacologic activation of NQO1 in these models successfully triggered ferroptosis and restored CD8^+^ T-cell-mediated immune infiltration. Moreover, NQO1 expression levels were proposed as a potential predictive biomarker for immunotherapy sensitivity. These findings suggest that the KEAP1–Nrf2–NQO1 axis represents a unique therapeutic vulnerability in immunotherapy-refractory HNC and warrants further investigation as a dual ferroptosis–immune-modulating target.

Taken together, these findings illustrate the complexity of ferroptosis resistance in HNC. A diverse network of regulatory mechanisms—including RNA modifications, viral proteins, methylation events, microenvironmental signals, and immune-evasive transcriptional feedback—converge on the Nrf2–SLC7A11–GPX4 axis to protect tumor cells. Notably, the KEAP1–Nrf2–NQO1 pathway not only promotes ferroptosis resistance but also impairs antitumor immunity, representing a dual barrier to effective treatment. Targeting these determinants could enhance the efficacy of ferroptosis-based therapies, particularly when combined with immune checkpoint inhibitors or redox-disrupting agents [7]. Future studies should focus on developing tumor-specific inhibitors and delivery systems to exploit these mechanisms safely and effectively in clinical settings [66,67].

Despite the growing interest in ferroptosis-targeted strategies, measuring ferroptosis in preclinical HNC models remains challenging due to the lack of specific biomarkers and the context-dependent variability of ferroptotic features. Reliance on surrogate markers such as lipid ROS or GPX4 loss, without corroborating cell death assays, may result in misinterpretation. Standardized approaches and validated ferroptosis reporters are urgently needed to improve reproducibility.

## 5. Ferroptosis Modulation as a Therapeutic Strategy in Head and Neck Cancer

**Figure 4:**
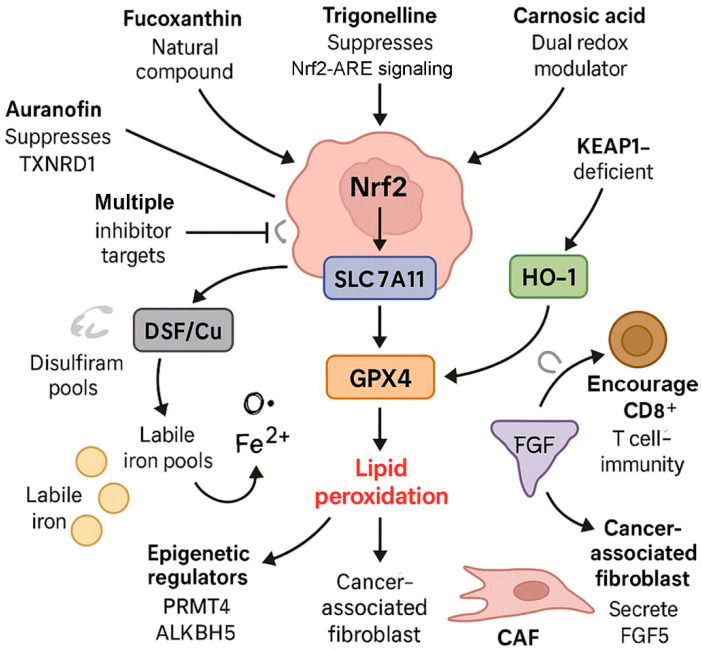
Ferroptosis modulation as a therapeutic strategy in head and neck cancer. Therapeutic interventions targeting the Nrf2–SLC7A11–GPX4 axis aim to overcome ferroptosis resistance in HNC. Compounds such as auranofin and trigonelline inhibit TXNRD1 or Nrf2, disrupting redox homeostasis. Carnosic acid acts as a dual redox modulator, while fucoxanthin directly downregulates antioxidant defenses. Disulfiram/copper (DSF/Cu) elevates labile iron pools, promoting ROS-mediated lipid peroxidation. Epigenetic regulators like PRMT4 and ALKBH5 enhance Nrf2 expression or stability. Tumor-derived signals (e.g., FGF5 from CAFs) activate HO-1 and suppress ferroptosis, but their inhibition restores lipid peroxidation and CD8^+^ T-cell immunity. These strategies collectively sensitize HNC to ferroptotic cell death and immune checkpoint blockade.

Building upon the molecular mechanisms outlined in the previous sections, which highlight how redox adaptation, epigenetic regulation, viral signaling, and tumor–stromal interactions contribute to ferroptosis resistance in HNC, this section focuses on therapeutic strategies designed to overcome these barriers. Pharmacologic induction of ferroptosis has gained substantial attention as a promising approach to combat treatment resistance in aggressive HNC subtypes, including hypopharyngeal and nasopharyngeal carcinomas. These malignancies often exhibit suboptimal responses to standard therapies such as platinum-based chemotherapy and immune checkpoint inhibitors, partly due to the Nrf2-driven upregulation of antioxidant and iron-buffering systems [68,69]. By targeting these protective pathways, either directly or indirectly, therapeutic interventions can sensitize resistant tumors to ferroptosis and potentially restore treatment efficacy (Figure 4 and Table 1) [51,70].

Several preclinical studies have demonstrated the feasibility of using ferroptosis inducers to inhibit tumor progression in HNC models. One such compound is auranofin, a gold-based inhibitor of TXNRD1, which promotes ferroptosis by disrupting thiol redox homeostasis [73]. In a study by Hsieh et al., auranofin not only suppressed NRF2 signaling but also enhanced CD8^+^ T-cell infiltration and improved responsiveness to anti-PD-1 checkpoint blockade in HNSCC [33]. Notably, TXNRD1 expression positively correlated with PD-L1 levels and was elevated in immunotherapy-resistant tumors. These findings support the rationale for dual targeting of TXNRD1 and Nrf2 to simultaneously trigger ferroptosis and immunogenic cells.

Fucoxanthin, a marine carotenoid, has also demonstrated potent ferroptosis-inducing properties [74,75]. Du et al. reported that fucoxanthin significantly downregulated GPX4, SLC7A11, and Nrf2 expression in SCC-25 tongue cancer cells while increasing intracellular ROS, malondialdehyde (MDA), and total iron content [71]. These changes led to mitochondrial damage and lipid peroxidation. Molecular docking studies showed strong binding affinities of fucoxanthin to Nrf2 and GPX4, suggesting direct inhibitory interactions. Moreover, reduced levels of GSH and superoxide dismutase (SOD) confirmed disruption of both redox and iron metabolism pathways.

Carnosic acid, a polyphenol derived from rosemary, was initially shown to protect against erastin-induced ferroptosis through activation of the Nrf2 transcriptional pathway [76,77]. More recently, however, carnosic acid has also been demonstrated to reverse cisplatin resistance in oral SCC by promoting ferroptosis, suggesting a context-dependent dual role in redox regulation and cell death control. Han et al. demonstrated that carnosic acid suppressed the Nrf2–HO-1–SLC7A11 axis in cisplatin-resistant CAL27-DDP and SCC9-DDP cells, which otherwise exhibited elevated GSH levels and reduced lipid peroxidation [72]. Carnosic acid increased ROS and lipid peroxide levels, effects that were reversed by the ferroptosis inhibitor liproxstatin-1. Reintroduction of Nrf2 activity attenuated the effects of carnosic acid, confirming the central role of Nrf2 suppression in restoring ferroptotic vulnerability.

Notably, disulfiram/copper (DSF/Cu) complexes have been found to promote ferroptosis by increasing intracellular labile iron levels and enhancing lipid peroxidation [48]. However, activation of the Nrf2–HO-1 pathway served as a compensatory mechanism that limited ferroptotic cell death. Inhibition of this pathway significantly amplified the cytotoxic effects of DSF/Cu in both in vitro and xenograft models, underscoring the therapeutic potential of combining Nrf2 suppression with ferroptosis-inducing agents.

An additional ferroptosis-modulating compound of interest is trigonelline, a naturally occurring alkaloid that has demonstrated the ability to reverse therapy resistance in HNC through suppression of the Nrf2–ARE signaling axis. In earlier studies by Roh and colleagues, trigonelline was shown to sensitize cisplatin-resistant HNC cells to artesunate-induced and GPX4 inhibitor-induced ferroptosis, respectively [27,28]. Mechanistically, trigonelline attenuated the transcriptional activity of Nrf2, reduced the expression of antioxidant enzymes including HO-1, and restored lipid peroxidation in resistant cells [78]. These effects were observed both in vitro and in vivo, indicating its dual role in impairing redox adaptation and enhancing susceptibility to ferroptosis in otherwise refractory tumors. Importantly, trigonelline demonstrated selective toxicity toward cancer cells without harming normal tissues, underscoring its therapeutic potential as an Nrf2-targeting adjuvant in ferroptosis-based strategies for HNC. Given its capacity to counteract resistance to multiple ferroptosis inducers—including artesunate and RSL3—trigonelline may serve as a broad-spectrum enhancer of ferroptotic vulnerability, particularly in tumors characterized by Nrf2 pathway activation.

An additional ferroptosis-related target in HNC is NAD(P)H quinone dehydrogenase 1 (NQO1), a direct transcriptional target of NRF2. Recent findings have revealed that in KEAP1-deficient and immunotherapy-resistant HNSCC models, induction of NQO1 can trigger ferroptosis while simultaneously initiating antitumor immune activation. Yuan et al. demonstrated that pharmacologic targeting of NQO1 in these tumors not only overcame ferroptosis resistance but also restored CD8+ T-cell infiltration and enhanced sensitivity to immune checkpoint inhibitors [34]. Importantly, elevated NQO1 expression was proposed as a predictive biomarker for immunotherapy responsiveness in KEAP1-mutant contexts. These data suggest that selective NQO1 modulation represents a dual-action therapeutic strategy capable of dismantling redox-driven immune evasion.

Targeting the tumor microenvironment presents an additional opportunity for modulating ferroptosis in human cancers [79,80]. Liu et al. reported that FGF5 secreted by CAFs activated FGFR2 in NPC cells, which in turn upregulated the Keap1–Nrf2–HO-1 pathway and blocked cisplatin-induced ferroptosis [30]. Neutralization of FGF5 or FGFR2 restored ferroptosis and improved drug sensitivity, highlighting the therapeutic potential of disrupting stromal contributions to redox adaptation.

Emerging evidence also emphasizes the role of epigenetic and post-transcriptional regulators of Nrf2 in cancer [81]. Ye et al. showed that the m^6^A RNA demethylase ALKBH5 stabilized Nrf2 mRNA by promoting its interaction with IGF2BP2 in hypopharyngeal SCC [29]. Silencing ALKBH5 reduced Nrf2 levels and sensitized cells to ferroptosis, suggesting that modulation of RNA methylation and RNA-binding protein networks may be a novel therapeutic approach.

Extracellular vesicles (EVs) have likewise been implicated in ferroptosis resistance [82,83]. Li et al. demonstrated that platelet-derived EVs enriched in integrin β3 (ITGB3) enhanced metastatic capacity and ferroptosis evasion in NPC cells [31]. Mechanistically, ITGB3 activated the MAPK/ERK/ATF4/Nrf2 signaling axis, leading to SLC7A11 stabilization and suppression of lipid peroxidation. These findings support targeting EV biogenesis or uptake to disrupt intercellular activation of ferroptosis resistance pathways.

Collectively, these mechanistic insights strongly support the therapeutic relevance of ferroptosis modulation in HNC. Several strategies have been proposed to selectively promote ferroptotic cell death in resistant tumor subtypes. One direct approach involves pharmacological inhibition of GPX4 or SLC7A11, two enzymes essential for detoxifying lipid peroxides and maintaining redox balance [84,85]. Inhibiting these molecules leads to the accumulation of oxidized phospholipids, culminating in ferroptotic death. Another strategy centers on disrupting the upstream regulators of Nrf2, a master transcription factor that enhances ferroptosis resistance [20,39]. Epigenetic and post-translational modulators such as PRMT4, which stabilizes Nrf2 by inhibiting its ubiquitination, and TXNRD1, which supports Nrf2 transcriptional activity through thiol redox regulation, represent viable targets [32,33].

In summary, the natural compound trigonelline has been shown to suppress Nrf2–ARE signaling and enhance the efficacy of ferroptosis inducers such as artesunate and GPX4 inhibitors in cisplatin-resistant HNC cells [27,28], highlighting its potential as a tumor-selective Nrf2 antagonist. Additionally, targeting downstream NRF2 effectors such as NQO1 in KEAP1-mutant tumors can trigger ferroptosis while simultaneously activating antitumor immunity, expanding the scope of dual-action therapeutic opportunities [34]. Inhibiting these regulators has been shown to decrease GPX4 and SLC7A11 expression and restore ferroptosis sensitivity in preclinical HNC models. In parallel, increasing the intracellular LIP is also effective in potentiating ferroptosis. This can be achieved through induction of ferritinophagy, which liberates stored iron, or modulation of HO-1, which releases free iron during heme degradation [48,71,72]. However, the effect of HO-1 is context-dependent and must be tightly regulated to avoid paradoxical cytoprotection [45]. Exogenous cues from the tumor microenvironment also play a critical role in ferroptosis evasion. Factors such as FGF5 released by CAFs, integrin β3-enriched EVs from activated platelets, and EBV-driven oncogenic signals have all been shown to activate the Keap1–Nrf2 pathway, thereby amplifying the antioxidant response and suppressing ferroptotic signaling [55,83]. Interfering with these external activators may help dismantle the redox shield that protects HNC cells from oxidative cell death. Finally, combination therapies that incorporate ferroptosis inducers with existing modalities such as chemotherapy, radiotherapy, or immune checkpoint blockade have shown synergistic effects in overcoming resistance. For example, ferroptosis induction enhances CD8^+^ T-cell infiltration and downregulates PD-L1 expression, re-sensitizing tumors to anti–PD-1 therapy [33].

While multiple studies support the regulatory role of Nrf2 in ferroptosis evasion, most findings are derived from in vitro models or preclinical settings. The lack of robust clinical correlation limits direct therapeutic translation. Moreover, mechanistic overlaps between ferroptosis and other forms of cell death remain underexplored. Despite the promise of these approaches, a major clinical challenge remains: how to selectively induce ferroptosis in tumor cells without causing collateral damage to normal tissues [70]. Given the physiological role of Nrf2 in protecting healthy cells from oxidative and electrophilic stress, systemic inhibition of this pathway may lead to adverse effects [61]. Therefore, future efforts must focus on developing tumor-specific ferroptosis regulators or delivery systems—such as nanoparticle-based carriers, ligand-directed inhibitors, or gene-editing platforms—that allow for precise targeting of malignant cells while sparing normal tissues [86,87].

## 6. Conclusions and Future Perspectives

The interplay between ferroptosis and the Nrf2 signaling pathway represents a critical determinant of therapeutic vulnerability and resistance in HNC. As this review has outlined, ferroptosis—a form of regulated cell death driven by iron-catalyzed lipid peroxidation—offers a mechanistically distinct and potentially targetable pathway for overcoming the treatment resistance that characterizes many HNC subtypes. Central to this process is the transcription factor Nrf2, which modulates a broad network of genes involved in redox balance, GSH metabolism, lipid detoxification, and iron storage. While Nrf2 activation serves as a protective response under physiological conditions, its aberrant stabilization in cancer cells—whether through somatic mutations, oncogenic stress, viral infection, or microenvironmental signals—confers resistance to ferroptosis and conventional therapies alike. The resulting upregulation of SLC7A11, GPX4, HO-1, and related antioxidant enzymes shields tumor cells from lethal oxidative damage, supporting survival even under therapeutic pressure.

Recent preclinical studies have provided compelling evidence that disrupting Nrf2 or its downstream effectors can restore ferroptosis sensitivity in HNC. Agents such as fucoxanthin, carnosic acid, and disulfiram/copper complexes have demonstrated the ability to induce ferroptotic death by suppressing Nrf2-related pathways. In addition, molecular modulators such as PRMT4, TXNRD1, ALKBH5, and extracellular vesicle-derived proteins have been identified as upstream regulators of Nrf2 activity. The NRF2 transcriptional target NQO1 has also emerged as a downstream effector that mediates ferroptosis resistance and immune evasion in KEAP1-deficient tumors; pharmacologic targeting of NQO1 not only restores ferroptotic sensitivity but also enhances antitumor immune responses. Inhibition of these targets not only reactivates ferroptosis but also sensitizes resistant tumors to chemotherapy and immunotherapy, offering opportunities for synergistic combination treatments.

Despite these promising advances, several challenges remain. The dual role of Nrf2 as both a tumor promoter and a critical protector of normal tissue homeostasis complicates its direct therapeutic targeting. Systemic suppression of Nrf2 may lead to adverse effects in non-malignant tissues, particularly those with high basal oxidative stress. To overcome this limitation, there is an urgent need to develop tumor-selective strategies that can inhibit Nrf2 activity specifically in malignant cells. This may include the use of targeted delivery systems such as nanoparticles, ligand-conjugated inhibitors, or gene-editing technologies that exploit tumor-specific metabolic or genetic vulnerabilities.

In parallel, identifying predictive biomarkers of ferroptosis susceptibility is essential for successful clinical translation. For example, evaluating GPX4 expression levels, lipid peroxidation status, or Nrf2 pathway activity in tumor biopsies could enable the stratification of patients who are most likely to benefit from ferroptosis-based interventions. Additionally, validating ferroptosis-targeting strategies in physiologically relevant models such as patient-derived organoids and xenografts will be crucial to assess efficacy, safety, and drug response heterogeneity before clinical application. In particular, alterations in NFE2L2, KEAP1, or SLC7A11 expression could serve as predictive indicators of ferroptosis resistance, with the potential for guiding patient selection in clinical trials. Moreover, elevated NQO1 expression may act as a surrogate biomarker for identifying KEAP1-mutant tumors that are both ferroptosis-resistant and immunotherapy-refractory. RNA sequencing or single-cell transcriptomics of HNC samples could reveal ferroptosis-prone subtypes, aiding in the development of personalized therapies that combine ferroptosis induction with immunomodulation or metabolic intervention.

Moreover, there is a growing recognition that ferroptosis can influence antitumor immunity. Preclinical data suggest that ferroptosis induction can enhance T-cell infiltration and reduce immune checkpoint molecule expression, thereby amplifying the efficacy of immunotherapy. Therefore, incorporating ferroptosis-inducing agents into current immunotherapeutic regimens may enhance tumor immunogenicity and overcome resistance to checkpoint blockade in HNC. Finally, dietary and metabolic influences—such as the availability of polyunsaturated fatty acids or LIP—may modulate tumor susceptibility to ferroptosis. Understanding how nutritional and systemic factors impact ferroptotic sensitivity could provide opportunities to optimize treatment outcomes through metabolic modulation. Recent studies emphasize the immunological implications of ferroptosis in shaping the tumor microenvironment, including the modulation of CD8^+^ T-cell infiltration and immunogenic cell death pathways. Ferroptosis-induced lipid peroxidation has been shown to release oxidized phospholipids and DAMPs (damage-associated molecular patterns) that promote T cell-mediated antitumor immunity [88,89].

Although most findings to date are preclinical, several ferroptosis-inducing strategies—especially those targeting Nrf2 and its downstream effectors—show promise for overcoming drug resistance and improving immunotherapy responses in HNC. Therapeutic strategies targeting ferroptosis in HNC remain at a nascent stage, but several challenges must be addressed, including tumor heterogeneity, the dual role of Nrf2, and the lack of reliable biomarkers for ferroptosis sensitivity. With advances in tumor-specific delivery systems and biomarker-driven stratification, these strategies may soon enter clinical evaluation. Incorporating artificial intelligence (AI)-driven models for predicting ferroptosis sensitivity and patient-specific redox profiles may also refine treatment planning and improve therapeutic outcomes. While AI application in this area is still in its infancy, emerging studies suggest that its integration could facilitate personalized medicine approaches in oncology [90]. Integration of computational tools and AI-guided molecular profiling may help identify patient subsets most likely to benefit from ferroptosis-inducing agents [91]. In the context of enhancing ferroptosis-inducing therapeutics, supramolecular approaches are gaining attention. Particularly, the supramolecular assembly of BODIPY-based systems has shown promise in improving photodynamic and photothermal efficiency, tissue penetration, and tumor-specific delivery [92]. Such methods may synergize with ferroptosis inducers through ROS amplification and lipid peroxidation enhancement. Clinical trials are needed to validate these findings and clarify therapeutic windows.

In conclusion, ferroptosis represents a compelling therapeutic avenue for HNCs that are resistant to conventional treatments. The Nrf2 pathway, long known for its antioxidant and cytoprotective roles, emerges as a central obstacle to ferroptotic cell death and a key mediator of therapy resistance. Continued investigation into the molecular underpinnings of ferroptosis, the development of tumor-specific Nrf2-targeting strategies, and the integration of ferroptosis inducers into multimodal treatment regimens may unlock new therapeutic possibilities for patients with advanced or refractory HNC.

## Figures and Tables

**Table 1:** Ferroptosis-modulating compounds and mechanisms in head and neck cancer.

Compound/Modulator	Mechanism of Action	HNC Subtype/Model	Therapeutic Insight	Reference(s)
Auranofin	Inhibits TXNRD1; suppresses Nrf2 signaling; enhances CD8^+^ T-cell infiltration	HNSCC (PD-L1^+^, resistant models)	Synergizes with anti-PD-1 therapy; ferroptosis and immunotherapy sensitization.	[33]
Fucoxanthin	Downregulates GPX4, SLC7A11, and Nrf2; increases ROS, MDA, and iron	SCC-25 (tongue SCC cells)	Induces mitochondrial damage and ferroptosis via redox and iron pathway disruption.	[71]
Carnosic acid	Inhibits the Nrf2–HO-1–SLC7A11 pathway; increases lipid peroxidation	CAL27-DDP, SCC9-DDP (cisplatin-resistant OSCC)	Reverses cisplatin resistance; promotes ferroptotic sensitivity in resistant OSCC cells.	[72]
Disulfiram/Cu (DSF/Cu)	Increases labile iron and lipid peroxidation; counteracted by Nrf2–HO-1 activation	OSCC cell lines; xenograft models	Nrf2 inhibition enhances DSF/Cu cytotoxicity and ferroptosis induction.	[48]
Trigonelline	Suppresses Nrf2–ARE signaling; reduces HO-1; restores lipid peroxidation	HNC cisplatin-resistant models	Enhances ferroptosis by sensitizing cells to artesunate and GPX4 inhibitors; tumor-selective redox modulation.	[27,28]
NQO1 (KEAP1-deficient context)	NRF2 target gene; induces ferroptosis; triggers antitumor immune activation	HNSCC (KEAP1-deficient and immunotherapy-resistant)	Overcomes ferroptosis and immune resistance via NQO1-mediated ferroptosis; potential biomarker for immunotherapy sensitivity.	[34]
FGF5/FGFR2 (CAF-derived)	Activates Keap1–Nrf2–HO-1 signaling; blocks ferroptosis	NPC (CAF co-culture and in vivo models)	Stromal signaling promotes redox resistance; targeting FGFR2 restores cisplatin-induced ferroptosis.	[30]
ALKBH5/IGF2BP2 axis	Stabilizes Nrf2 mRNA via m6A demethylation; suppresses ferroptosis	Hypopharyngeal SCC	RNA methylation-dependent ferroptosis resistance; epitranscriptomic therapeutic target.	[29]
Platelet EVs / ITGB3	Activates MAPK/ERK/ATF4/Nrf2; increases SLC7A11 and suppresses lipid peroxidation	NPC cell lines and xenograft models	EV-mediated intercellular ferroptosis evasion; targeting EV signaling restores redox imbalance	[31]

## Abbreviations

ALKBH5: AlkB homolog 5, RNA demethylase; ARE, antioxidant response element; ATF4, activating transcription factor 4; CAF, cancer-associated fibroblast; EBV, Epstein–Barr virus; EV, extracellular vesicles; FGF5, fibroblast-derived fibroblast growth factor 5; FGFR2, fibroblast growth factor receptor 2; FPN1, ferroportin; FTH1, ferritin heavy chain 1; GPX4, glutathione peroxidase 4; GSH, glutathione; HNC, head and neck cancer; HNSCC, head and neck squamous cell carcinoma; HO-1, heme oxygenase-1; IGF2BP2, insulin-like growth factor 2 mRNA-binding protein 2; ITGB3, integrin subunit beta 3; Keap1, Kelch-like ECH-associated protein 1; LIP, labile iron pool; m^6^A, N^6^-methyladenosine; MAPK, mitogen-activated protein kinase; MDA, malondialdehyde; NPC, nasopharyngeal carcinoma; NQO1, NAD(P)H quinone dehydrogenase 1; Nrf2, nuclear factor erythroid 2-related factor 2; PERK, protein kinase R-like ER kinase; PD-L1, programmed death-ligand 1; PRMT4, protein arginine N-methyltransferase-4; ROS, reactive oxygen species; SCC, squamous cell carcinoma; SLC7A11, solute carrier family 7 member 11; SOD; superoxide dismutase; system Xc^−^, cystine/glutamate antiporter; TXNRD1, thioredoxin reductase 1.
